# Distributions of straw-derived carbon in Mollisol’s aggregates under different fertilization practices

**DOI:** 10.1038/s41598-021-97546-3

**Published:** 2021-09-09

**Authors:** Zhuang Ge, Tingting An, Roland Bol, Shuangyi Li, Ping Zhu, Chang Peng, Yingde Xu, Na Cheng, Tingyu Li, Yihui Wu, Ninghui Xie, Jingkuan Wang

**Affiliations:** 1 Northeast Key Laboratory of Conservation and Improvement of Cultivated Land (Shenyang), Ministry of Agriculture, College of Land and Environment, Shenyang Agricultural University, Shenyang, 110866 Liaoning China; 2 Institute of Bio- and Geosciences, Agrosphere (IBG-3), Forschungszentrum Jülich GmbH, Wilhelm-Johnen-Straße, 52428 Jülich, Germany; 3 Institute for Biodiversity and Ecosystem Dynamics (IBED), University of Amsterdam, 1012 WX Amsterdam, The Netherlands; 4 Jilin Academy of Agricultural Sciences, Gongzhuling, 136100 Jilin China

**Keywords:** Biogeochemistry, Carbon cycle

## Abstract

Straw incorporation is an effective measure for increasing soil organic carbon (SOC) thereby improving soil quality and crop productivity. However, quantitative assessments of the transformation and distribution of exogenous carbon (C) in soil aggregates under various field fertilization practices have been lacking. In this study, we collected topsoil samples (0–20 cm) from three fertilization treatments (no fertilization control, CK; inorganic fertilizer, IF; inorganic fertilizer plus manure, IFM) at a 29-year long-term Mollisol experiment in Northeast China. We then mixed the soil samples with ^13^C-labeled maize straw (δ^13^C = 246.9‰), referred as CKS, IFS, and IFMS, and incubated them *in-situ* for 360 days. Initial and incubated soil samples were separated into four aggregate fractions (> 2, 1–2, 0.25–1, and < 0.25 mm) using the dry-sieving method, which counted 18%, 17%, 45%, and 21% (averages from the three initial soil samples), respectively. Organic C content was highest in 0.25–1 mm aggregate (6.9–9.6 g kg^−1^) prior to incubation, followed by > 2 mm aggregates (2.2–5.8 g kg^−1^), 1–2 mm aggregates (2.4–4.6 g kg^−1^), and < 0.25 mm aggregates (3.3–4.5 g kg^−1^). After 360-day incubation with straw incorporation, organic C content was 2.3–4.5 g kg^−1^, 2.9–5.0 g kg^−1^, 7.2–11 g kg^−1^ and 1.8–3.0 g kg^−1^ in > 2, 1–2, 0.25–1, and < 0.25 mm aggregates, respectively, with the highest in the IFMS treatment. Straw-derived C content was 0.02–0.05 g kg^−1^, 0.03–0.04 g kg^−1^, 0.11–0.13 g kg^−1^, and 0.05–0.10 g kg^−1^ in > 2, 1–2, 0.25–1, and < 0.25 mm aggregates, respectively. The relative distribution of straw-derived C was highest (40–49%) in 0.25–1 mm aggregate, followed by < 0.25 mm aggregates (21–31%), 1–2 mm aggregates (13–15%), and > 2 mm aggregates (9.4–16%). During the incubation, the relative distribution of straw-derived C exhibited a decrease in > 2 mm and 1–2 mm aggregates, but an increase in the < 0.25 mm aggregate. At the end of incubation, the relative distribution of straw-derived C showed a decrease in the 0.25–1 mm aggregate but an increase in the < 0.25 mm aggregate under the IFMS treatment. This study indicates that more straw-derived C would be accumulated in smaller aggregates over longer period in Mollisols, and combined inorganic and organic fertilization is an effective measure for C sequestration in Northeast China.

## Introduction

Soil organic carbon (SOC) is an important component of the global carbon (C) cycle, as it makes up generally two-thirds of the terrestrial C pool^1^. Even small changes in SOC stocks can have a substantial influence on atmospheric CO_2_ concentration^2^. On the other hand, SOC, as a key index of soil fertility, has a large influence on the maintenance of crop productivity due to its significant relative contribution to the overall properties of soils^1,3^. Hence, enhancing SOC storage through improved land management is crucial to sustainable agriculture and also mitigation of global warming^3^.

The formation and stability of soil aggregates is a key process for SOC sequestration^4^. Different sizes of aggregates have various characteristics, and the physical protection of soil aggregate controls the distribution and turnover of SOC^5^. Furthermore, straw incorporation is an efficient and economical agricultural practice to prevent soil degradation and improve SOC sequestration in agricultural systems^6^. Straw addition improves the formation of macroaggregates with a concomitant decline of microaggregates and increases organic C accumulation in various sizes of aggregate^7^. Moreover, fertilizer management is a global strategy to improve soil quality, increase crop productivity and restrain the decreasing of SOC^8^. Long-term application of mineral fertilizer and/or organic amendments could increase the SOC content and change the distribution of SOC in aggregates^9^. Therefore, it is important to quantitatively investigate that how fertilizer management strategy affects the dynamics of exogenous straw straw-derived C in soil aggregates.

In our previous study, we only separate two sizes of aggregate and investigate the dynamics of straw C incorporation into these two sizes of aggregates under a laboratory incubation condition^10^. More aggregate sizes should provide more detailed information about straw C incorporation in soil^11^. Besides, laboratory incubation is carried out under constant temperature and moisture conditions, while soil thermal and moisture environments in field experiments are largely changed during a year^12^. Research data are still needed to verify how exogenous straw C is transformed and stabilized, also for more sizes of aggregate in the field conditions than the previous study^10^. Such study outcomes will be much closer to the actual agricultural reality.

The Northeast China Plain is one of the most important food production regions in China, where Mollisol (US Soil Taxonomy) is the dominant soil type. This region occupies about 25% of national arable land and produces more than 30% of national foods as commodities, and thus has an important influence on China's food security^13,14^. However, in the past several decades, long-term unreasonable field management such as conventional tillage and crop straw removal or burning has led to serious agricultural problems, such as soil degradation, and substantial SOC losses, which results in a significant reduction in soil fertility^15,16^. Solving the problem of Mollisol degradation is an urgent task. To better understand the mechanism of transformation and distribution of straw C in soil aggregates affected by different fertilizer management strategies, we conducted an in-situ incubation experiment based on a long-term experiment of Mollisols. The objective of this study was to investigate the dynamics of transformation and distribution of crop straw C in soil aggregates under different fertilizer management strategies. Overall, this study will reveal the mechanism of exogenous straw C sequestration in soil aggregates and optimize fertilizer management strategies in the Mollisols region of Northeast China.

## Results

### Organic C content in soil aggregate

**Figure 1:**
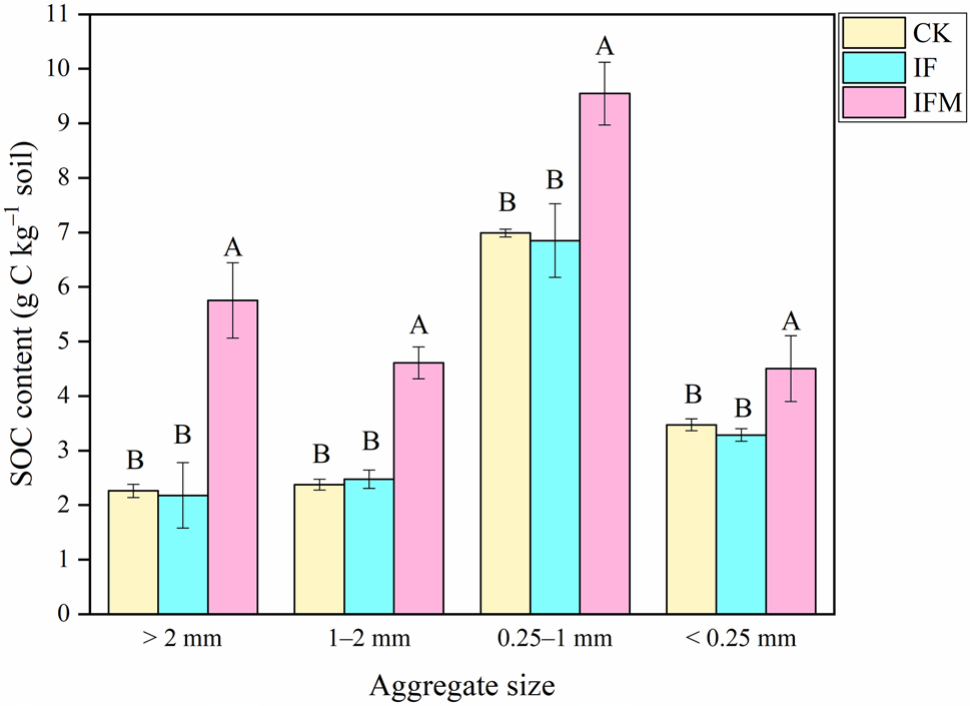
Effects of 29-year long-term fertilization on SOC content in soil aggregates. *Note*: The CK denotes the no fertilization control treatment, IF inorganic fertilizer treatment, and IFM inorganic fertilizer plus manure treatment. Vertical bars represent the standard error of the mean significant variations (SEM; n = 3). Values followed by different letters mean significant variations (*P* < 0.05) in SOC content among various fertilizer management strategies for the same aggregate size.

Organic C content was highest in 0.25–1 mm aggregate (6.9–9.6 g kg^−1^) prior to incubation, followed by > 2 mm aggregates (2.2–5.8 g kg^−1^), 1–2 mm aggregates (2.4–4.6 g kg^−1^), and < 0.25 mm aggregates (3.3–4.5 g kg^−1^) (Fig. 1). Compared to CK and IF treatments, organic C content in IFM treatment was averagely increased by 159% (> 2 mm aggregates), 90% (1–2 mm aggregates), 38% (0.25–1 mm aggregates) and 33% (< 0.25 mm aggregates).

**Figure 2:**
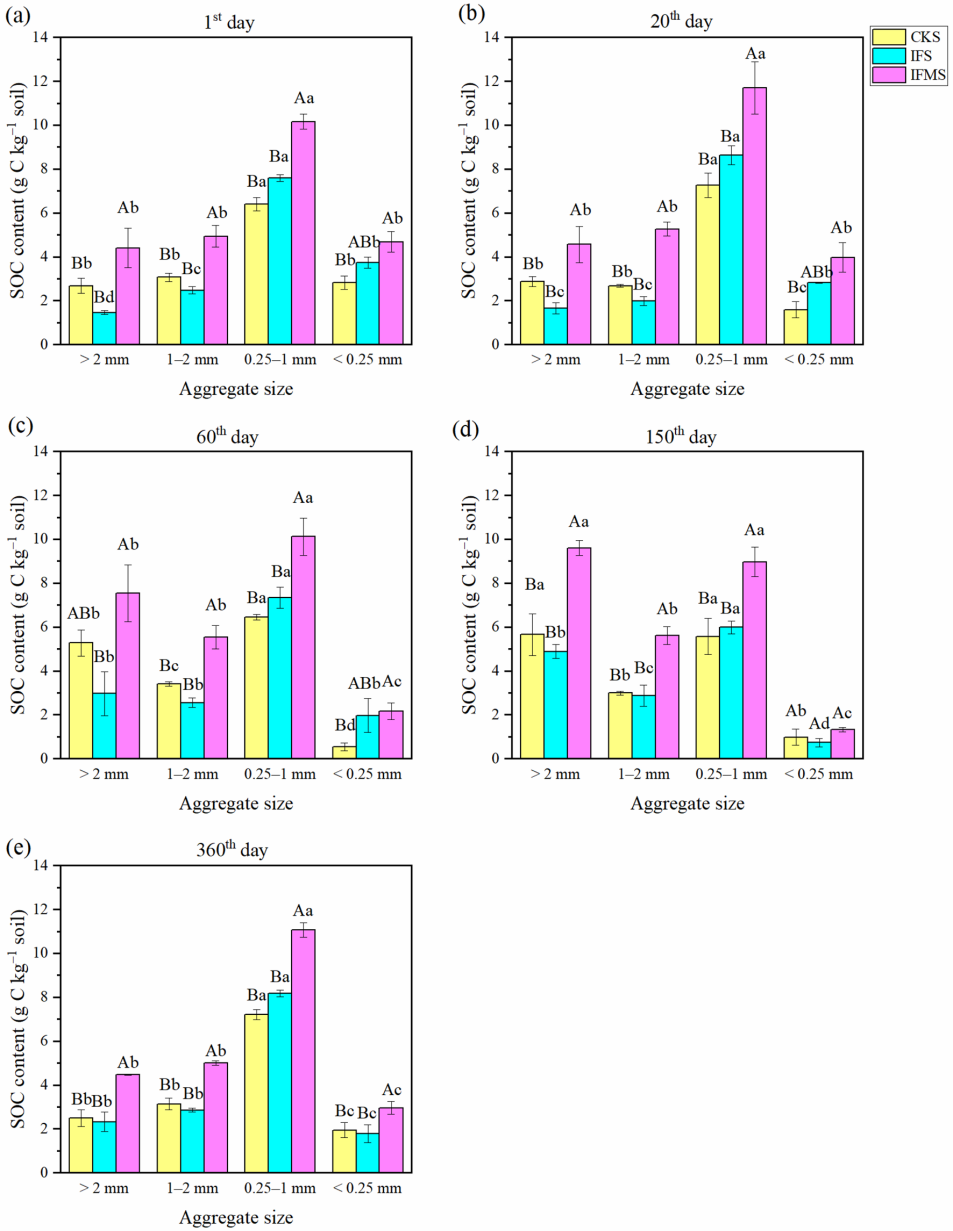
The SOC contents in aggregates under various fertilizer management strategies after adding straw. *Note*: Treatments including CKS (no fertilization control + straw), IFS (inorganic fertilizer + straw), IFMS (inorganic fertilizer plus manure + straw). Vertical bars represent the standard error of the mean significant variations (SEM; n = 3). Values followed by different letters (uppercase among various fertilizer management strategies and lowercase letters among various aggregate sizes) mean significant variations (*P* < 0.05) in SOC contents.

After straw addition, organic C content had a significant (*P* < 0.001) relationship with aggregate size and fertilizer management strategy (Table 1). Organic C content was 2.3–4.5 g kg^−1^, 2.9–5.0 g kg^−1^, 7.2–11 g kg^−1^ and 1.8–3.0 g kg^−1^ in > 2, 1–2, 0.25–1, and < 0.25 mm aggregate, respectively (Fig. 2). IFMS treatment has the highest organic C content during the whole incubation period. Organic C contents of all aggregate fractions in IFMS treatment were, on average, 79% and 84% larger than those in CKS and IFS treatments.

**Table 1:** Analysis of variance (ANOVA) results on interactions the effects of treatment, aggregate size, incubation period on SOC content and straw-derived C content.

Variation	df	SOC content	Straw-derived C content
Treatment (T)	2	***	ns
Aggregate size (A)	3	***	***
Incubation period (D)	4	ns	***
T × A	6	***	ns
T × D	5	ns	*
A × D	12	***	***
T × A × D	24	ns	ns

* and *** indicate significant differences at *P* < 0.05 and *P* < 0.001 level, respectively. ns represents no statistical significance at the *P* < 0.05 level.

### Distributions of straw-derived C in soil aggregates

**Figure 3:**
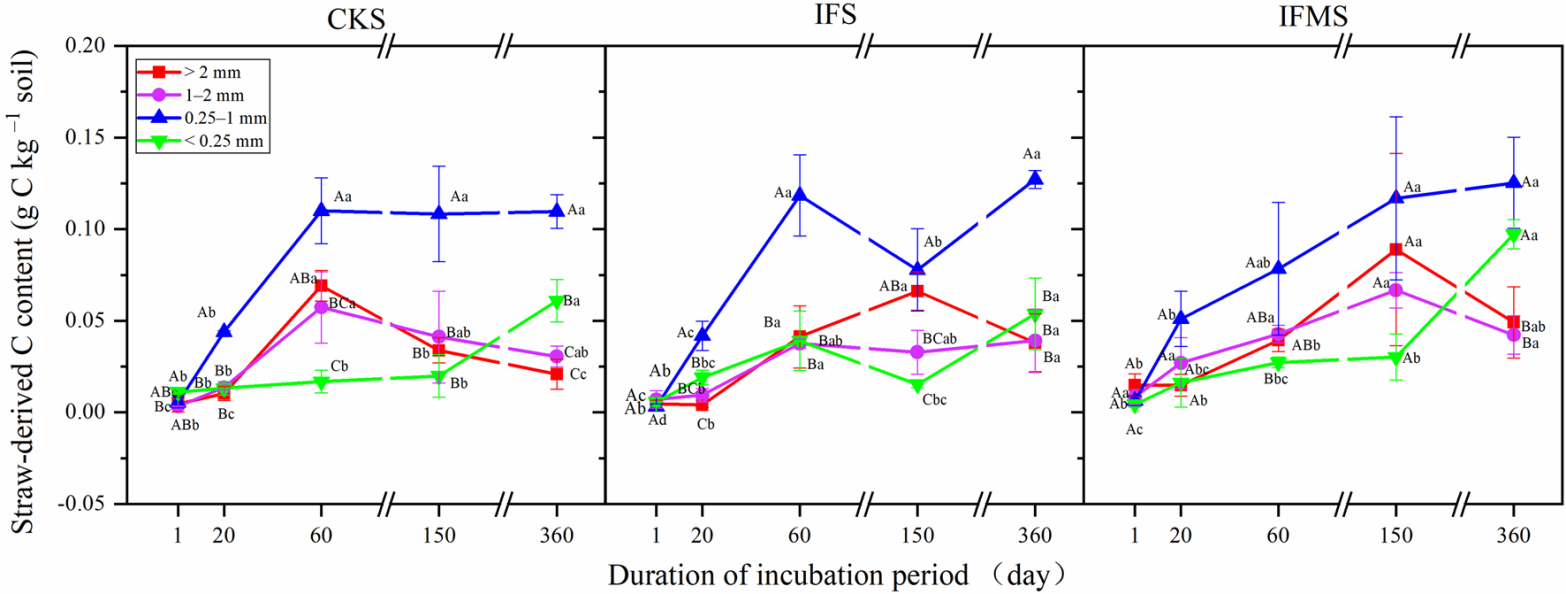
The straw-derived C contents in aggregates under different fertilizer management strategies. *Note*: Treatments including CKS (no fertilization control + straw), IFS (inorganic fertilizer + straw), IFMS (inorganic fertilizer plus manure + straw). Vertical bars represent the standard error of the mean significant variations (SEM; n = 3). Values followed by different letters (uppercase among various aggregate sizes and lowercase among various incubation periods) mean significant variations (*P* < 0.05) in straw-derived C contents.

Straw-derived C content had a significant (*P* < 0.001) relationship with aggregate size, incubation period and their interactions (Fig. 3 and Table 1). The straw-derived C content in 0.25–1 mm aggregate was highest, i.e., 0.11 g kg^−1^, 0.13 g kg^−1^, and 0.13 g kg^−1^ in the treatments of CKS, IFS, and IFMS treatments, respectively (Fig. 3). In CKS treatment, straw-derived C content in > 2 mm and 1–2 mm aggregate was the highest on day 60. Straw-derived C content in < 0.25 mm aggregate was enhanced by 200% from day 150 to 360. In IFS treatment, straw-derived C content in < 0.25 mm aggregate was increased by 150%, but that in > 2 mm aggregate was decreased by 44% from day 150 to 360. In IFMS treatment, straw-derived C content in < 0.25 mm aggregate was increased by 233% from day 150 to 360, while that in > 2 mm aggregate and 1–2 mm aggregate was decreased by 44% and 43% from day 150 to 360, respectively.

### Relative distribution percentage of straw-derived C to soil aggregates

**Figure 4:**
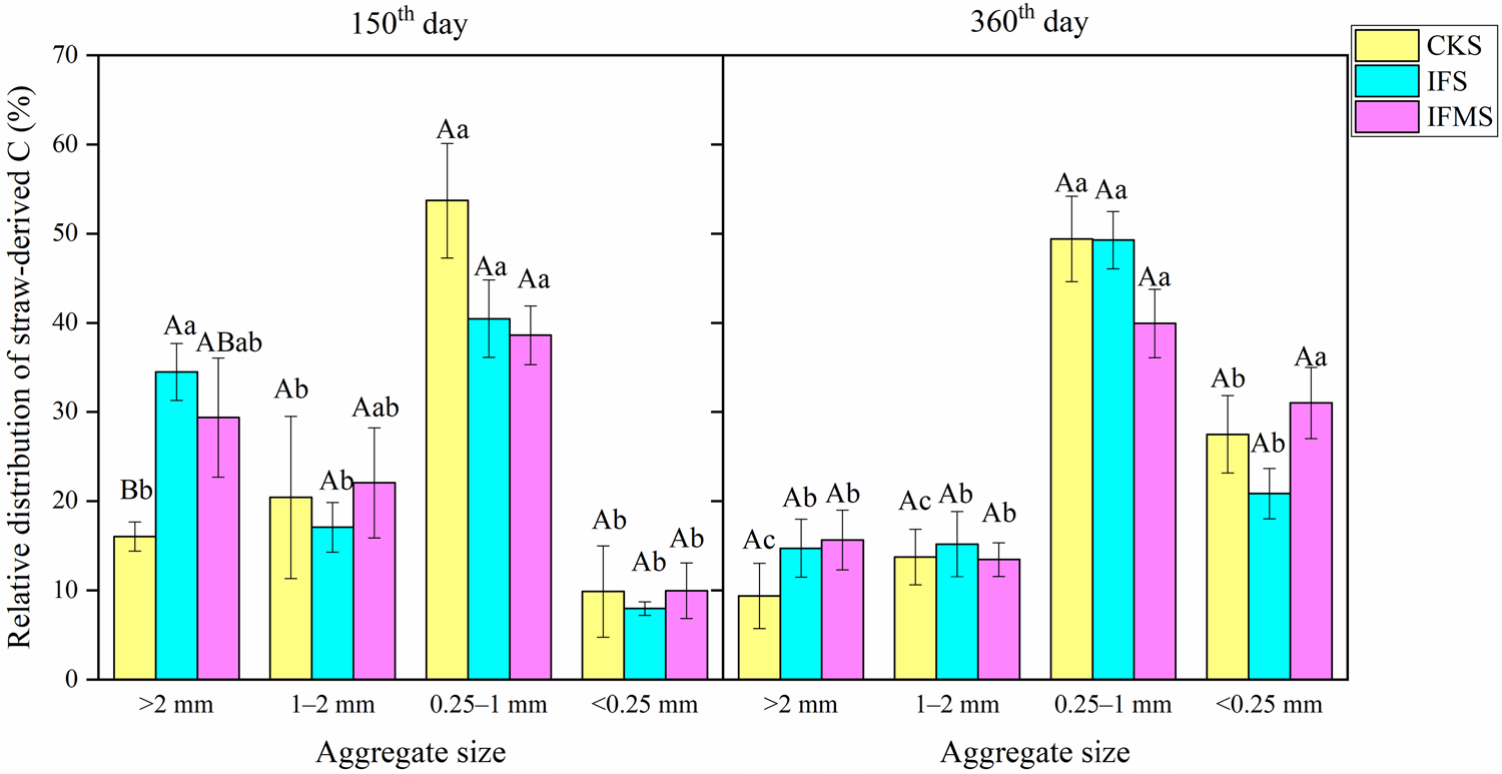
The relative distribution of straw-derived C in soil aggregates (%). *Note*: Treatments including CKS (no fertilization control + straw), IFS (inorganic fertilizer + straw), IFMS (inorganic fertilizer plus manure + straw). Vertical bars represent the standard error of the mean significant variations (SEM; n = 3). Values followed by different letters (uppercase among various fertilizer management strategies and lowercase letters among various aggregate sizes) mean significant variations (*P* < 0.05) in the relative distribution of straw-derived C in soil aggregates (%).

The relative distribution of straw-derived C to 0.25–1 mm aggregate was 54%, 40%, and 39% in CKS, IFS, and IFMS treatments on day 150, respectively (Fig. 4). About one-half of straw-derived C was distributed to 0.25–1 mm aggregate in CKS and IFS treatments, and 40% of straw-derived C was incorporated into 0.25–1 mm aggregate in IFMS treatments on day 360. Besides, on day 150, the relative distribution of straw-derived C to > 2 mm aggregate and 1–2 mm aggregate was higher than that to < 0.25 mm aggregate in three treatments, while there was an opposite trend on day 360.

## Discussion

### Effects of long-term fertilization on organic C in aggregates

Previous studies have reported that there is an increase of organic C in aggregate under long-term application of inorganic fertilizer without organic amendments in various soils, e.g., Ultisol^17^, Anthrosol^18^, and Vertisol^19^. For example, compared to no fertilizer application, long-term (21-year) application of inorganic fertilizer increased organic C content by 49, 42, and 40% in the aggregate fractions of sizes > 2, 0.25–2, and < 0.25 mm in Anthrosol, respectively^18^. However, we found that long-term (29-year) inorganic fertilization alone had no significant effect on organic C content in aggregates in Mollisol (Fig. 1). This result is mainly due to Mollisol is the most fertile soil, it has higher microbial biomass and activity^20^. Long-term application of inorganic fertilizer alone accelerates organic C mineralization and losses in Mollisol, making it inadequate to sustain C levels^21^.

Long-term straw incorporation may have different effects on organic C content in various sizes of aggregates between different types of soils. For example, in Ultisol, the largest organic C content occurs in 1–2 mm aggregate^22^, but in Aridisol, the largest organic C content occurs in > 2 mm aggregate^23^. The discrepancy in these results may be due to the differences in soil properties and climatic conditions. Aridisol is characterized by aridity, it may accumulate calcium carbonate and has a very low concentration of organic matter^23^. Acidification is one of the most serious problems associated with agricultural production in Ultisol, limiting the availability of nutrients, and increasing nutrients and SOC losses^17^. As a result, Aridisol and Ultisol have lower organic C content and fertility than Mollisol^24^. In C-poor soils, straw input will be preferentially digested by microorganisms to supplement organic C in larger aggregate^25^.

While our study showed that the largest organic C content was in the 0.25–1 mm aggregate, the relative distribution of straw-derived C exhibited a decrease in > 2 mm and 1–2 mm aggregates, but an increase in < 0.25 mm aggregates during the incubation. There was also evidence that the increase of organic C content was greatest in the 0.053–0.25 mm aggregate in Mollisol after long-term (> 20-year) straw incorporation^26^. These results suggested that more straw-derived C would be accumulated in smaller aggregates over longer period.

### Effects of fertilizer management on the sequestration of straw-derived C in soil aggregates

The transformation of straw-derived C in soil aggregates is influenced by fertilizer management strategies^27^. In our study, straw-derived C content in > 2 mm aggregate decreased on day 60 in the CKS treatment, which was earlier than that in the IFS and IFMS treatments (on day 150) (Fig. 3). This result indicated that newly-added straw-derived C might be preferentially decomposed in no fertilizer soil than in fertilized soil due to the availability of C source in no fertilizer soil is limited^10^. The application of inorganic fertilizer plus manure may have improved soil fertility and enhance soil microbial biomass C, thus straw-derived C is beneficial for the propagation of soil microbes and may therefore facilitate the conversion of C in IFMS treatment^28^. Meanwhile, microbes preferentially utilized straw-derived C by the metabolic and respiratory processes in larger aggregates, and thus the larger aggregate is more sensitive to the addition of exogenous fresh C than the other aggregates^29^. Moreover, the straw-derived C content in < 0.25 mm aggregates reached the maximum at the end of the incubation period (on day 360) in all fertilizer treatments in our study (Fig. 3). However, a previous study reported that under a lab incubation condition, the largest straw-derived C content in < 0.25 mm aggregates are founded at the short-term (on day 45) or mid-term (on day 135) of incubation period in the CKS and IFMS treatments, and at the end of the incubation period (on day 360) in the IFS treatment^10^. This difference could be due to the various experimental conditions impact the transformation of straw-derived C. Under the lab condition, the soil has a constant moisture content, inorganic fertilizer and manure addition promotes the turnover of SOC due to nitrogen addition, thus the content of straw-derived C in < 0.25 mm aggregate reaches its maximum first in a relatively short period in the no fertilizer treatment^30^. However, under the field condition, drying and wetting may influence the dynamics of straw-derived C and reduce straw-derived C stabilization in three treatments, thus the contents of straw-derived C in < 0.25 mm aggregate reach their maximum at the end of incubation period^31^. Similar results also occurred in other studies that were conducted in Alfisol and Aquic Inceptisol under field condition^32,33^. These results suggested that different incubation conditions could affect straw decomposition, it could change the redistribution of C in soils due to releasing extractable C, growing biomass, and enhancing C mineralization in the soil with different fertilizers^34^*.*

Straw-derived C could transform among various sizes of aggregates^35^. Our study showed that straw-derived C transformed from the larger aggregates to the smaller aggregates in three treatments. Similar results also occur in other types of soils, for example, in Alfisol, straw-derived C is mainly stored in larger soil aggregates in the short-term (45 days) experiment, but it is shifted into being stored in smaller aggregates after a 3-year incubation^36^; in Anthrosol, exogenous fresh straw C in microaggregates has generally persisted longer than the C in macroaggregates^35^. These results suggested that different sizes of aggregates have distinct physical protection capacities for fresh exogenous C^37^. Macroaggregates represent the point where exogenous fresh straw-derived C enters the system of aggregates and have lower physical protection capacity, while the microaggregates represent the final stage in the organic C transformation^35^ and it could promote the stabilization of exogenous C^38^. As a result, straw-derived C could be redistributed from macroaggregate to microaggregae^39,40^.

### Effects of fertilizer management on the relative distribution of straw-derived C in soil aggregates

The relative distribution of straw-derived C to aggregate fractions was strongly affected by fertilization. The relative distribution of straw-derived C sequestrated into 0.25–1 mm aggregate in CKS treatment was much higher than in IFS and IFMS treatments on day 150 (Fig. 4). This result also confirmed that exogenous fresh C could be quickly incorporated into the soil with lower nutrients. With the decomposition of straw, exogenous straw-derived C gradually accumulated from larger to smaller aggregates in IFS treatment on day 360. It indicated the newly-added straw-derived C may be preferentially used for microbial processes, which leads to the breakdown of larger aggregates in IFS treatment^41^. Besides, the relative distribution of straw-derived C in 0.25–1 mm aggregate was the highest among all the aggregate fractions in our study, which indicated that straw-derived C is mainly occluded within 0.25–1 mm aggregate and thus is “protected”^42^. The previous study of Alfisol also observed that the largest proportion of straw-derived C is sequestrated in 0.25–1 mm aggregate^33^. However, straw-derived C is mostly sequestrated in > 2 mm aggregate in Aquic Inceptisol^32^. Aquic Inceptisol is characterized by a high content of sand particles and a low level of organic C, therefore, straw-derived C is preferentially preserved in larger aggregate^43^. It suggested that the sequestration processes of exogenous C in different soil aggregates could be related to initial SOC content, and exogenous C would be sequestrated mostly in smaller aggregate in the soil with a higher SOC content.

The dynamic changes of the relative distribution of straw-derived C in < 0.25 mm aggregate over time in our study indicated that straw-derived C would be gradually entered into microaggregate. A similar result also occurs in the Aquic Inceptisol^32^, it indicated that < 0.25 mm aggregate has lower reactive minerals^44^, and straw-derived C is hardly incorporated within this size of aggregate in a short-term incubation period. With the incubation period increases, straw-derived C was gradually sequestrated in < 0.25 mm aggregate. Therefore, our result confirmed that microaggregate would act as temporary storage sites for straw-derived C, and 0.25–1 mm aggregates are the main sites for accumulation and stabilization of SOC during straw decomposition^45^.

## Conclusion

This in-situ mini-plot field experiment was conducted to investigate the dynamics of transformation and distribution of straw-derived C in soil aggregates under different fertilizer management strategies in the Mollisols. Long-term application of inorganic fertilizer alone had no significant effect on the organic C content of soil aggregates compared with no fertilizer treatment. After 360-day incubation with straw incorporation, organic C content and straw-derived C content in aggregates were the highest in the soil with inorganic fertilizer plus manure practice. During the incubation, the relative distribution of straw-derived C exhibited a decrease in > 2 mm and 1–2 mm aggregates, but an increase in the < 0.25 mm aggregate. At the end of incubation, the relative distribution of straw-derived C showed a decrease in the 0.25–1 mm aggregate but an increase in the < 0.25 mm aggregate under the IFMS treatment. This study indicates that more straw-derived C would be accumulated in smaller aggregates over longer period in Mollisols, and combined inorganic and organic fertilization is an effective measure for carbon sequestration in Northeast China.

## Methods

### Study site description

The long-term field experiment site used in this study was located at the Jilin Academy of Agricultural Sciences at Gongzhuling, Jilin, northeast China (43°30′N, 124°48′E, and 200 m above sea level). The region was established in 1990, has a typical continental monsoon climate with a mean annual temperature of 4–5 °C and a mean annual precipitation of 400–600 mm^46^. The soil was a Mollisols (classified as a Luvic Phaeozem, FAO) with 39% sand, 30% silt, and 31% clay at the beginning of the experiment^47^. Three application strategies included in this study consisted of (1) unfertilized control (CK), (2) balanced inorganic fertilizers at 165 kg N ha^−1^,82.5 kg P_2_O_5_ ha^−1^, and 82.5 kg K_2_O ha^−1^ (IF), (3) balanced inorganic fertilizers at 50 kg N ha^−1^, 82.5 kg P_2_O_5_ ha^−1^, and 82.5 kg K_2_O ha^−1^ plus manure (115 kg N ha^−1^) at a rate of 2.3 × 10^4^ kg ha^−1^ (IFM)^48^. The manure is pig manure and generally left outside in the yard for about 4–5 months, then applied in autumn after maize harvesting in the IFM plots each year^46,48^. Urea was used as nitrogen (N) fertilizer; triple superphosphate was used as phosphorus (P) fertilizer, and potassium sulfate was used as potassium (K) fertilizer^10^. The organic C and N content of the manure were approximately 112 g kg^−1^ and 5.0 g kg^−1^, respectively; the δ^13^C of manure had an average value of − 21.59‰^49^. The soil properties at the start of the experiment were as follows: pH 6.7, organic C 28.0 g kg^−1^, total N content 1.9 g kg^−1^, and total P content (as P_2_O5) 0.6 g kg^−1^^50^.

### In-situ field experiment design

The mini-plot experiment was not undertaken at the main long-term field site itself, but in a nearby field to avoid any presence of ^13^C-labeled straw influencing future natural abundance soil ^13^C measurements. Two soil pits of the following dimensions (length × width × height = 0.9 m × 0.6 m × 0.3 m) were therefore dug in a nearby field for the mini-plot experiment. Two Polyvinyl Chloride (PVC) material boxes (length × width × height = 0.9 m × 0.6 m × 0.6 m) of similar dimensions to the pit were then inserted vertically into field pits on May 5, 2018, i.e., the boxes were 0.3 m above the ground level to avoid any impacts by other soil in the field. The boxes were not closed at the bottom to allow for drainage. Each box consisted of nine equal sections, allowing three random replicates of the three treatments under consideration (CK, IF, and IFM). The topsoil layer (0–20 cm) was taken from each fertilization treatment of the long-term field experiment and individually passed in the field through a 5 cm sieve to remove crop roots and rocks. The SOC contents in the soil treatment before mixing were 14.95 ± 0.09 g kg^−1^ (CK), 14.98 ± 0.09 g kg^−1^ (IF), and 25.17 ± 0.23 g kg^−1^ (IFM). The δ^13^C value in the soil treatment before mixing was − 18.8 ± 0.0‰ (CK), − 19.4 ± 0.0‰ (IF), and − 19.1 ± 0.1‰ (IFM) (More details can be found in Table S1). The ^13^C-labeled maize straw were mature maize plants pulse-labeled using ^13^CO_2_ for four times over a growing season according to the procedure^51^. The ^13^C-labeled maize straw used aboveground part and had a total C 356 g kg^−1^ and δ^13^C 246.9‰. The ^13^C-labeled maize straw was cut in the size of 0.5–1.0 cm. The method of straw incorporation was based on the concept of full straw incorporation, i.e., where all straw after harvest is plowed back into the soil. In one box all 9 compartments consisted of the soil mixed with ^13^C-labeled straw (CKS, IFS, IFMS), the other box only contained soil (CK, IF, IFM). We added 36 g ^13^C-labeled straw to 15.84 kg of soil per compartment (equivalent to 2.3 g straw kg^−1^ soil). No plants were grown in all the boxes during the experimental period. Before completely filling the sections, we first added only soil from three treatments in each section box to the bottom 10 cm (20–30 cm depth). Subsequently, the upper 20 cm was filled with soil, part of the soils from the three treatments were mixed homogenously with ^13^C-labeled straw. Soil samples were collected at the depth of 0–20 cm for five times: on May 6, 2018 (1 day), May 25, 2018 (20 days), May 25, July 4, 2018 (60 days), October 2, 2018 (150 days), April 30, 2019 (360 days). We collected the soil samples in different locations in the compartment each time. Soil samples were put in the sealed bag, and we kept the soil in its original state after transferring them in the sealed bags. Samples were not stacked and thus not potentially squeezed. We stored the samples in the low-temperature incubator, transported them to the laboratory, prior to dry sieving immediately undertaken when arriving in the laboratory.

### Soil aggregate fractionation

The soil samples (500 g each) were air-dried according to the procedure^52^, field-moist clods of soil were cool-dried at 4 °C environmental condition until soil moisture contents reaching on 8%, at which point soil were able to be sieved to separate the aggregates. Soil samples were sieved through the 2 mm, 1 mm and 0.25 mm meshes on the Vibratory Sieve Shaker AS 200 (Retsch, Germany) for 2 min, amplitude 1.5 mm. The following four aggregate fractions were obtained including large macroaggregates (> 2 mm), medium macroaggregates (1–2 mm), small macroaggregates (0.25–1 mm), and microaggregates (< 0.25 mm). The proportion of soil aggregates under different fertilizer treatments with and without straw during the incubation period can be found in Table S2.

### Calculation and statistical analysis

The total SOC contents and δ^13^C values were measured with an elemental analyzer (Elementar Vario PYRO cube, Germany) coupled to an isotope ratio mass spectrometer (Isotope Ratio Mass Spectrometer, IsoPrime100, Germany). The δ^13^C value (‰) was expressed relative to Vienna Pee Dee Belemnite (VPDB) standard. The proportions of maize-derived C (f_maize_, %) in the soil with maize straw were estimated by the following^53^:

1 $$ {\text{f}}_{{{\text{maize}}}} = {{\left( {\updelta ^{{{13}}} {\text{C}}_{{{\text{sample}}}} {-}\updelta ^{{{13}}} {\text{C}}_{{{\text{soil}}}} } \right)} \mathord{\left/ {\vphantom {{\left( {\updelta ^{{{13}}} {\text{C}}_{{{\text{sample}}}} {-}\updelta ^{{{13}}} {\text{C}}_{{{\text{soil}}}} } \right)} {\left( {\updelta ^{{{13}}} {\text{C}}_{{{\text{straw}}}} {-}\updelta ^{{{13}}} {\text{C}}_{{{\text{soil}}}} } \right)}}} \right. \kern-\nulldelimiterspace} {\left( {\updelta ^{{{13}}} {\text{C}}_{{{\text{straw}}}} {-}\updelta ^{{{13}}} {\text{C}}_{{{\text{soil}}}} } \right)}} $$

where δ^13^C_sample_ represents the δ^13^C value of SOC in the treatment with maize straw at a certain time; δ^13^C_soil_ represents the δ^13^C value of the initial soil; and δ^13^C_straw_ represents the δ^13^C value of the initial maize straw.

Thus, the content of maize straw-derived C at a certain time in the treatments with maize straw was calculated as follows^54^.

2 $$ {\text{C}}_{{{\text{straw}}}} = {\text{C}}_{{{\text{sample}}}} \times {\text{f}}_{{{\text{maize}}}} $$

Statistical analyses were performed using the PASW Statistics software version 18.0 (IBM Crop., Armonk, NY, USA). All the results were shown means of three replicates with standard deviation. The data were subjected to one-way ANOVA and Duncan tests to evaluate the effects of different fertilization treatments and incubation periods. Significant differences were reported at the *P* < 0.05 level. Three-factor analyses of variance (ANOVAs) using the PASW Statistics software version 18.0 to evaluate the interactions of treatment, aggregate size, and incubation period on SOC content and straw-derived C content. Overall, three levels of significance were defined and indicated by asterisks. The Graphical work was performed using Origin Pro 2019 (OriginLab, Northampton, MA, USA).

## Supplementary Information

Supplementary Information.
