# Supplementary material for: Distributions of straw-derived carbon in Mollisol’s aggregates under different fertilization practices
Source: Sci Rep. 2021 Sep 9;11:17899. doi: 10.1038/s41598-021-97546-3 (PMC8429711; doi:10.1038/s41598-021-97546-3)
Supplement: Supplementary file 1 — Supplementary Information. [file 41598_2021_97546_MOESM1_ESM.docx]

**Supplementary material**

**Table S1**

Soil basic properties of different fertilizer management strategies in 2018.

| Treatment | SOC (g kg^–1^) | δ^13^C (‰) | TN (g kg^–1^) | C/N ratio | AP (mg kg^–1^) | AK(mg kg^–1^) |
| --- | --- | --- | --- | --- | --- | --- |
| CK | 14.95±0.09 b | –18.78±0.01 a | 1.42±0.06 c | 10.57±0.37 a | 15.32±0.75 c | 65.04±3.18 c |
| IF | 14.98±0.09 b | –19.37±0.03 c | 1.55±0.01 b | 9.67±0.05 b | 20.90±0.70 b | 81.42±1.40 b |
| IFM | 25.17±0.23 a | –19.13±0.06 b | 2.60±0.02 a | 9.67±0.02 b | 80.66±2.56 a | 137.37±3.87 a |

Note: The CK denotes no fertilization control treatment, IF inorganic fertilizer treatment, IFM inorganic fertilizer plus manure treatment. SOC denotes soil organic carbon, TN denotes total nitrogen, C/N denotes the ratio of SOC and total nitrogen, AP denotes available phosphorous, AK denotes available potassium. Different lower-case letters mean significant differences (*P* < 0.05) in various fertilizer management strategies.

**Table S2**

The proportion (%) of aggregates under different fertilizer treatments with and without straw during the incubation period.

| Incubation period （day） | Treatment | > 2 mm | | 1-2 mm | | 0.25-1 mm | | < 0.25 mm | |
| --- | --- | --- | --- | --- | --- | --- | --- | --- | --- |
|  |  | no straw | add straw | no straw | add straw | no straw | add straw | no straw | add straw |
| 1 | CK | 14.3 | 17.8 | 15.7 | 19.8 | 47.4 | 43.8 | 22.5 | 18.7 |
| 1 | IF | 15.1 | 8.7 | 15.5 | 13.3 | 47.9 | 53.8 | 21.5 | 24.2 |
| 1 | IFM | 23.0 | 17.9 | 19.7 | 19.7 | 38.6 | 42.1 | 18.7 | 20.3 |
| 20 | CK | 17.4 | 19.5 | 18.7 | 19.4 | 56.0 | 50.4 | 7.8 | 10.6 |
| 20 | IF | 12.8 | 10.8 | 15.1 | 14.1 | 59.8 | 57.0 | 12.3 | 18.1 |
| 20 | IFM | 26.0 | 18.0 | 19.5 | 19.3 | 44.1 | 46.8 | 10.4 | 15.9 |
| 60 | CK | 30.2 | 34.1 | 20.8 | 22.6 | 42.1 | 39.8 | 6.8 | 3.5 |
| 60 | IF | 22.9 | 21.1 | 17.2 | 17.1 | 48.3 | 49.0 | 11.6 | 12.8 |
| 60 | IFM | 35.5 | 30.2 | 22.6 | 20.8 | 34.7 | 40.8 | 7.2 | 8.3 |
| 150 | CK | 44.6 | 37.5 | 22.5 | 20.4 | 30.3 | 36.1 | 2.7 | 6.1 |
| 150 | IF | 47.9 | 34.6 | 21.3 | 20.1 | 28.4 | 40.6 | 2.4 | 4.7 |
| 150 | IFM | 55.1 | 38.5 | 22.1 | 21.7 | 20.3 | 34.7 | 2.4 | 5.1 |
| 360 | CK | 19.2 | 16.6 | 21.4 | 21.1 | 48.6 | 49.1 | 10.7 | 13.2 |
| 360 | IF | 19.1 | 15.9 | 19.5 | 18.1 | 51.5 | 53.9 | 10.0 | 12.0 |
| 360 | IFM | 27.9 | 19.4 | 24.0 | 21.5 | 40.6 | 46.8 | 7.5 | 12.3 |

Note: The CK denotes no fertilization control treatment, IF inorganic fertilizer treatment, IFM inorganic fertilizer plus manure treatment.

**Table S3**

The content of SOC (g kg^-1^ soil) in soil aggregate without straw residue under different fertilizer management strategies.

| Treatment | | Incubation period (day) | > 2 mm | 1-2 mm | | 0.25-1 mm | | < 0.25 mm | |  |
| --- | --- | --- | --- | --- | --- | --- | --- | --- | --- | --- |
|  | 1 | | 2.26±0.12 | | 2.38±0.10 | | 6.99±0.07 | | 3.47±0.11 | |
|  | 20 | | 2.77±0.26 | 2.87±0.26 | | 8.16±0.37 | | 1.15±0.13 | |  |
| CK | 60 | | 4.65±0.46 | 3.13±0.30 | | 6.22±0.46 | | 1.03±0.23 | |  |
|  | 150 | | 6.65±0.89 | 3.25±0.18 | | 4.39±0.68 | | 0.42±0.02 | |  |
|  | 360 | | 2.92±0.19 | 2.94±0.18 | | 7.06±0.12 | | 1.58±0.21 | |  |
|  | 1 | | 2.18±0.60 | 2.47±0.17 | | 6.85±0.67 | | 3.29±0.11 | |  |
|  | 20 | | 1.90±0.31 | 2.09±0.09 | | 8.23±0.21 | | 1.78±0.59 | |  |
| IF | 60 | | 3.22±0.06 | 2.40±0.10 | | 6.76±0.17 | | 1.76±0.18 | |  |
|  | 150 | | 6.64±0.51 | 3.42±0.23 | | 4.21±0.42 | | 0.34±0.08 | |  |
|  | 360 | | 2.94±0.33 | 2.87±0.23 | | 7.67±0.58 | | 1.47±0.21 | |  |
|  | 1 | | 5.76±0.69 | 4.61±0.29 | | 9.55±0.58 | | 4.50±0.60 | |  |
|  | 20 | | 6.79±1.17 | 4.86±0.24 | | 11.07±1.22 | | 2.57±0.28 | |  |
| IFM | 60 | | 9.06±1.76 | 5.42±0.25 | | 8.67±1.18 | | 1.72±0.69 | |  |
|  | 150 | | 12.53±0.13 | 5.23±0.18 | | 5.05±0.39 | | 0.56±0.06 | |  |
|  | 360 | | 6.74±0.48 | 5.93±0.39 | | 9.16±0.48 | | 1.94±0.22 | |  |

Note: The CK denotes no fertilization control treatment, IF inorganic fertilizer treatment, IFM inorganic fertilizer plus manure treatment.

**
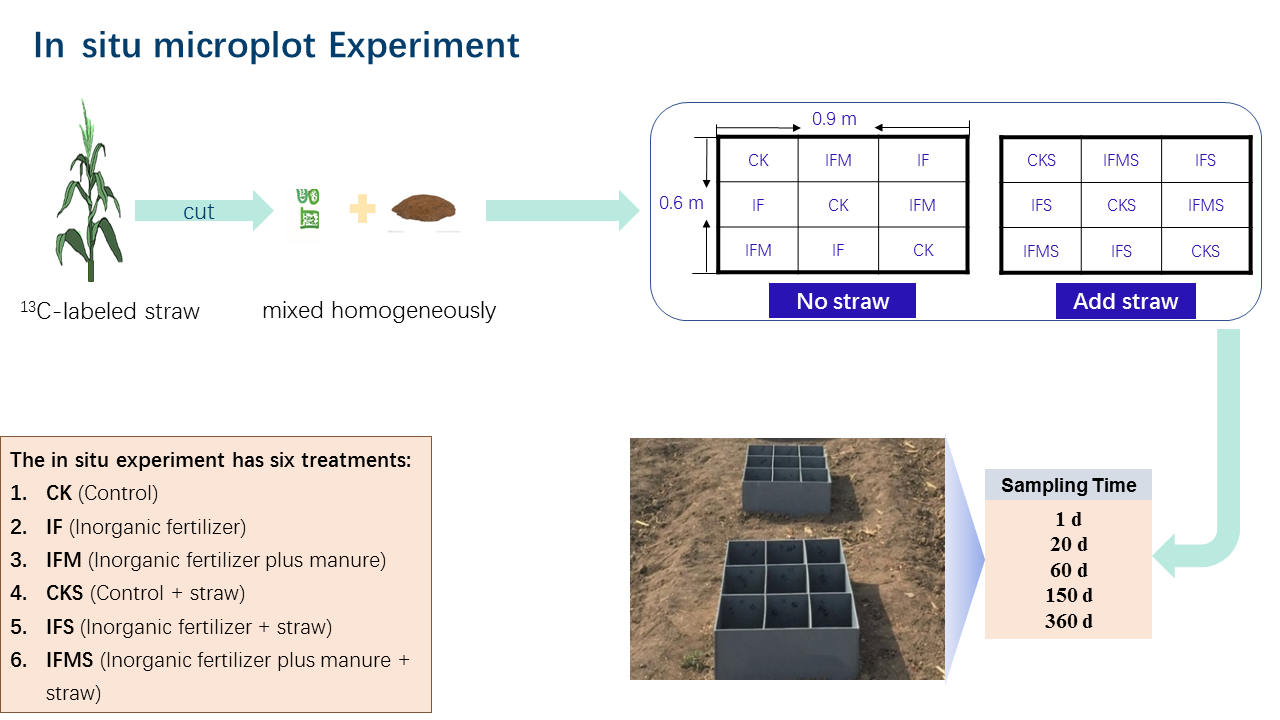
**

**Figure S1 The Schematic diagram of the in situ microplot experiment.**
